# Akt: A Double-Edged Sword in Cell Proliferation and Genome Stability

**DOI:** 10.1155/2012/951724

**Published:** 2012-03-15

**Authors:** Naihan Xu, Yuanzhi Lao, Yaou Zhang, David A. Gillespie

**Affiliations:** ^1^Division of Life Science, Graduate School at Shenzhen, Tsinghua University, Shenzhen 518055, China; ^2^School of Pharmacy, Shanghai University of Traditional Chinese Medicine, Cai Lun Lu 1200, Shanghai 201203, China; ^3^The Beatson Institute for Cancer Research, Garscube Estate, Switchback Road, Glasgow G61 1BD, Scotland, UK; ^4^College of Medical, Veterinary and Life Sciences, University of Glasgow, Glasgow G12 8QQ, UK

## Abstract

The Akt family of serine/threonine protein kinases are key regulators of multiple aspects of cell behaviour, including proliferation, survival, metabolism, and tumorigenesis. Growth-factor-activated Akt signalling promotes progression through normal, unperturbed cell cycles by acting on diverse downstream factors involved in controlling the G1/S and G2/M transitions. Remarkably, several recent studies have also implicated Akt in modulating DNA damage responses and genome stability. High Akt activity can suppress ATR/Chk1 signalling and homologous recombination repair (HRR) via direct phosphorylation of Chk1 or TopBP1 or, indirectly, by inhibiting recruitment of double-strand break (DSB) resection factors, such as RPA, Brca1, and Rad51, to sites of damage. Loss of checkpoint and/or HRR proficiency is therefore a potential cause of genomic instability in tumor cells with high Akt. Conversely, Akt is activated by DNA double-strand breaks (DSBs) in a DNA-PK- or ATM/ATR-dependent manner and in some circumstances can contribute to radioresistance by stimulating DNA repair by nonhomologous end joining (NHEJ). Akt therefore modifies both the response to and repair of genotoxic damage in complex ways that are likely to have important consequences for the therapy of tumors with deregulation of the PI3K-Akt-PTEN pathway.

## 1. Akt Family Kinases

As one of the most versatile kinase families, Akt (also known as PKB) serine-threonine kinases function as critical regulators of cell survival, proliferation, metabolism, and migration. Deregulation of Akt kinases is frequently associated with human diseases such as cancer and diabetes. Three isoforms of Akt have been identified in mammals: Akt1, Akt2, and Akt3 [1–3]. The Akt isoforms share approximately 80% amino acid identity and are thought to have similar primary substrate specificity [4]. They are widely expressed in various tissues; Akt1 is most abundant in brain, heart, and lung, whereas Akt2 is predominantly expressed in skeletal muscle and embryonic brown fat. Akt3 is mainly expressed in kidney, brain, and embryonic heart [3, 5–7]. Findings from Akt isoform-specific knockout mice suggest that Akt family kinases are likely to have distinct biological functions *in vivo*. Thus, Akt1 knockout mice are smaller than littermate controls and show increased rates of apoptosis in some tissues, reflecting the role of Akt1 in cell survival [8, 9]. By contrast, Akt2 null mice develop type 2 diabetes and impaired glucose utilization, suggesting that Akt2 function is more specific for the insulin receptor signaling pathway [10, 11]. The precise role of Akt3 is less clear, however, mice lacking Akt3 display impaired brain development [12].

All three Akt isoforms share a conserved N-terminal pleckstrin homology (PH) domain, a central kinase domain, and a C-terminal regulatory domain which contains the hydrophobic motif, a characteristic of the cAMP-dependent protein kinase A/protein kinase G/protein C (AGC) superfamily of protein kinases [13]. In response to various growth factors and cytokines, Akt activity is modulated downstream of phosphatidylinositol 3 (PI3) kinase via a multistep process. Activated PI3 kinase localizes to the cytoplasmic face of the plasma membrane, where it converts PIP2 (phosphatidylinositol 4,5-bisphosphate) to PIP3 (phosphatidylinositol 3,4,5-trisphosphate). PIP3 functions to activate downstream signaling components by recruiting proteins containing PH domains to the plasma membrane, such as Akt and PDK1 kinases [14–17]. Once recruited to the plasma membrane, Akt is activated by site-specific phosphorylation at residues Thr308 and Ser473. PDK1 phosphorylates Thr308 within the activation T-loop of the catalytic domain [18]. Ser473 within the carboxyl terminal hydrophobic domain can be phosphorylated by mammalian target of rapamycin complex 2 (mTORC2) [19]; however, other molecules, including integrin-linked kinase (ILK) and mitogen-activated protein kinase-activated protein linase-2 (MAPKAPK2), have also been reported to phosphorylate this residue [20, 21].

Once activated, Akt phosphorylates numerous substrates throughout the cell to regulate multiple cellular events and processes. Akt can also be activated in a PI3K-independent manner. For example, cAMP elevating agents have been shown to activate Akt through PKA [22, 23], whilst Ca^2+^/calmodulin-dependent kinase can directly phosphorylate and activate Akt *in vitro *[24, 25]. Several nonkinase interactors such as Hsp90, Hsp27, Tcl1, Geb10, and Ft1 have also been described to positively regulate Akt catalytic activity [13].

Negative regulation of PI3K/Akt pathways is mainly accomplished by PTEN (phosphatase and tensin homologue deleted on chromosome 10). PTEN was originally identified as a tumor-suppressor gene and is frequently mutated in a wide variety of solid tumors, including endometrial, breast, prostate carcinomas, and glioblastomas [26–28]. As a dual lipid and protein phosphatase, the primary physiological target of PTEN is considered to be the PI3K/Akt pathway [29–31]. PTEN specifically catalyses dephosphorylation of the 3′ phosphate of the inositol ring in PIP3, resulting in the biphosphate product PIP2 and inhibition of Akt activity [32]. Inactivating mutations or loss of PTEN expression leads to a permanent increase in the basal level of PI3K/Akt signaling, generally resulting in increased cell proliferation and resistance to apoptosis.

Recently, a family of novel protein phosphatases, namely, PHLPP (PH domain and leucine-rich repeat protein phosphatase), have been identified as important regulators of Akt kinases and protein kinase C (PKC) [33–35]. Two isoforms, PHLPP1 and PHLPP2, directly dephosphorylate Ser473 and therefore inactivate Akt. It has been shown that PHLPP differentially terminates Akt signaling by regulating distinct Akt isoforms. PHLPP2 dephosphorylates Akt1 and Akt3, whereas PHLPP1 is specific for Akt2 and Akt3 [35]. Several lines of evidence suggest that PHLPP functions as a tumor suppressor. PHLPP expression is commonly lost in cancer, including colon, breast, ovarian, Wilms tumors, and prostate cancer [33, 36, 37]. Overexpression of PHLPP1 in a glioblastoma cell line inhibits tumor growth in xenografted nude mice [34], and decreased expression of PHLPP has been linked to the metastatic potential of 21T breast cancer cells [38]. Codeletion of PHLPP1 and PTEN is strongly associated with metastatic prostate cancer and tightly correlated to deletion of p53 and PHLPP1, suggesting the role of PHLPP as a prostate tumor suppressor [37].

## 2. Role of Akt in Normal Cell Cycle Progression

**Figure 1:**
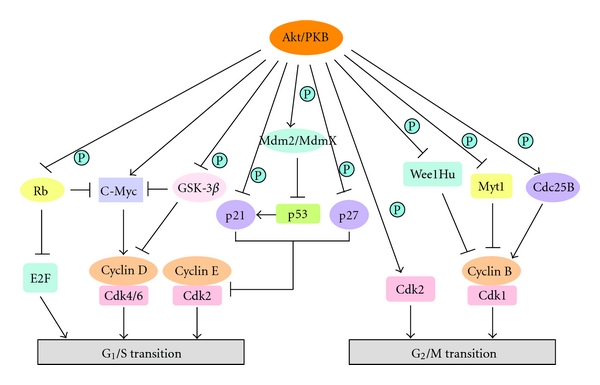
Role of Akt in normal cell cycle progression. Activated Akt kinase modulates the function of numerous proteins involved in cell cycle progression at the G1/S and G2/M transitions, either by direct phosphorylation of the target proteins themselves, or indirectly, by regulating protein expression levels. Please see text for additional explanation.

Remarkable progress has been made in determining how Akt promotes cell cycle progression. Activated Akt kinase modulates the function of numerous substrates related to cell cycle progression at the G1/S and G2/M transitions, either by direct phosphorylation of the target proteins themselves or, indirectly, by regulating protein expression levels. These are discussed in detail in the following and depicted in Figure 1.

### 2.1. Mechanisms of Akt Regulation of G1/S Progression

Addition of mitogens to quiescent (G0) mammalian cells rapidly triggers a number of biochemical signalling cascades, including the PI3K/Akt pathway, to promote cell growth through two key enzymes mTOR and p70S6K [39–42]. As depicted in Figure 1, growth-factor-mediated Akt activation increases the transcription of c-Myc, a strong promoter of cell cycle progression, causing cells to exit G0 both by inducing the expression of D-type cyclins and suppressing the expression of multiple negative cell cycle regulators such as p21^Cip1^, p27^Kip1^, and p15^INK4b^ [43, 44]. Akt also controls the stability of c-Myc and cyclin D1 indirectly *via* its downstream substrate, GSK-3*β*. GSK-3*β* phosphorylates c-Myc at Thr58, which appears to be required for ubiquitin-dependent proteolysis [45, 46]. Cyclin D1 is phosphorylated by GSK-3*β* at Thr286, a modification that induces nuclear-cytoplasmic translocation of cyclin D1 leading to ubiquitin-dependent proteolysis in the cytoplasm [47, 48]. Furthermore, PI3K/Akt is also necessary and sufficient to induce E2F transcriptional activity in T cells through phosphorylating and deactivating Rb [49].

Another important function of Akt in G1/S progression is positive regulation of mid- and late-G1-phase cyclin/Cdk activity *via *phosphorylation and inactivation of Cdk inhibitors, including p21^Cip1^ and p27^Kip1^. Akt phosphorylates p21^Cip1^ at Thr145, within its nuclear localization sequence (NLS), to recruit p21^Cip1^ from the nucleus to the cytoplasm and suppress its inhibitory effect on cell cycle progression [50]. Akt-mediated T145 phosphorylation also prevents p21^Cip1^ forming a complex with PCNA to inhibit DNA replication and decreases its binding to Cdk2/Cdk4 and attenuates its Cdk2 inhibitory activity [51]. In addition to Thr145, Ser146 is also phosphorylated by Akt, a modification that significantly increases p21^Cip1^ protein stability and protects cells from taxol-induced apoptosis [52]. Importantly, p21^Cip1^ protein induced by Akt does not inhibit cyclin E-Cdk2 but rather promotes the assembly and activation of cyclin D1-Cdk4 complex, which controls G1 to S phase progression and enhances cell cycling [52]. p21^Cip1^ elevation in tumors correlates with enhanced survival and chemoresistance [53], indicating that stabilization of p21^Cip1^ by Akt would be beneficial for tumor growth.

As with p21^Cip1^, the expression, localization, and protein stability of p27^Kip1^, are controlled by Akt-dependent phosphorylation. Akt is known to downregulate p27^Kip1^ transcription by phosphorylation-dependent inhibition of the Forkhead family of transcription factors [54]. Akt directly binds to and phosphorylates p27^Kip1^ at three residues, Ser10, Thr187, and Thr198 [55–58]. Ser10 phosphorylation could increase the nuclear export of p27^Kip1^ through binding to CRM1, but it is not sufficient to promote the cytoplasmic localization of p27^Kip1^ because an S10D mutant remains in the nucleus in G0/G1 cells [59]. Thr157 is within the nuclear localization sequence (NLS), and phosphorylation by Akt favours cytoplasmic accumulation of p27^Kip1^ [55–57, 60]. When forced to localize to the cytoplasm, p27^Kip1^ is less efficient in binding and inhibiting nuclear cyclin E-Cdk2 kinase activity and thus halting cell cycle progression [55–57]. Phosphorylation of C-terminal Thr198 by Akt promotes 14-3-3 binding and cytoplasmic localization of p27^Kip1^ [58]. Remarkably, cytoplasmic mislocalization of p27^Kip1^ has been observed in various human tumors [61, 62]. In up to 40% of primary human breast cancers, cytoplasmic p27^Kip1^ correlates with Akt activation, indicating that Akt-mediated cytoplasmic mislocalization of p27^Kip1^ may be critical in the development of human cancers [55].

Interestingly, recent studies revealed that the protein stability of p27^Kip1^ is controlled by Akt-dependent phosphorylation of Skp2 [63, 64]. Skp2 is a key component of SCF (Skp1/Cul-1/F-box) E3 ubiquitin ligase; it binds to p27^Kip1^ and targets it for ubiquitination and degradation [65–68]. Akt interacts with and directly phosphorylates Skp2 at Ser72, which triggers SCF complex formation and E3 ligase activity [63]. In addition, phosphorylation by Akt promotes the cytoplasmic localization of Skp2 and protects Skp2 from APC-Cdh1-mediated degradation [64]. High levels of active Akt correlates with the cytosolic accumulation of Skp2 in aggressive breast and prostate cancer patients, providing a rationale to develop specific Akt inhibitors as efficient anticancer drugs [64].

At the end of G_1_ phase, a series of safeguards ensure that the DNA is intact and the cell is ready to initiate DNA replication. DNA damage and other stresses can activate the tumor suppressor p53, which binds to DNA and up-regulates the expression of several genes involved in cell cycle control, DNA repair, senescence, angiogenesis, and apoptosis [69, 70]. In normal cells p53 is maintained at very low levels, and Mdm2 targets p53 for ubiquitination and allows export of p53 from the nucleus to the cytoplasm where proteolysis takes place [71, 72]. In response to growth factor stimulation, active Akt binds to and phosphorylates Mdm2 at Ser166 and Ser186 to enhance protein stability and facilitate the function of Mdm2 to promote p53 ubiquitination [73, 74]. Akt phosphorylation of Mdm2 is also necessary for the translocation of Mdm2 from the cytoplasm to the nucleus. Dominant-negative Akt inhibits nuclear entry of Mdm2, thus increasing the cellular level of p53 and transcriptional activity [75]. A close homologue of Mdm2, MdmX, has also been shown to be an Akt target. Unlike Mdm2, MdmX does not possess E3 ubiquitin ligase activity; however, it binds to and stabilizes Mdm2. The heterocomplex of Mdm2-MdmX is more efficient for p53 ubiquitination [76, 77]. Akt also phosphorylates MdmX at Ser367, which generates a 14-3-3 binding site and leads to the stabilization of the Mdm2-MdmX complex [78]. Thus, oncogenic Akt is able to functionally inactivate p53 by regulating Mdm2 and MdmX stability.

### 2.2. Mechanisms of Akt Regulation of G2/M Progression

It has been reported previously that Akt activity is high in the G2/M phase of the cell cycle in epithelial cells [79]. Akt activity protects cells from apoptosis during the G2/M transition and is necessary for efficient entry to mitosis during unperturbed cell cycles [79, 80]. As shown in Figure 1, Akt regulates the G2/M transition through direct phosphorylation of both Cdk1 activators and inhibitors, suggesting that the PI3K/AKT pathway could function to promote mitosis [81, 82].

As one of the three human members of Cdc25 family of dual-specificity phosphatases, Cdc25B plays an important role in the control of the activity of Cdk1/cyclin B [83]. The function of Cdc25B is tightly linked to its intracellular localization [84]. During the G2/M transition, Cdc25B shuttles between the nucleus and the cytoplasm as a result of the opposing actions of the nuclear localization (NLS) and nuclear export signals (NES) in association with 14-3-3 family members [81]. Akt-mediated Ser353 phosphorylation regulates the cytoplasmic accumulation of Cdc25B and contributes to mitotic entry by promoting its inactivation and access to specific substrates [82]. The Cdk1 inhibitor Wee1Hu (also known as Wee1A) is an Akt substrate in mammalian cells [85]. Akt binds to and phosphorylates Wee1Hu at Ser642 within its C-terminal catalytic domain during late S and G2 phase. Ser642 phosphorylation does not affect the kinase activity; however, it promotes cytoplasmic localization of Wee1Hu through binding to 14-3-3*θ* [85]. As a result, coexpression of Akt together with Wee1Hu and 14-3-3*θ* overcomes the G2/M arrest induced by Wee1Hu alone [85]. Another Cdk1 inhibitor Myt1 has also been shown to be a substrate for Akt in oocytes from the starfish *Asterina pectinifera *[86]. Akt phosphorylates Myt1 at Ser75 and severely reduces Myt1-mediated Tyr15 Cdk1 phosphorylation resulting in the activation of cyclin B-associated Cdk1 kinase activity [86]. Microinjection of nonphosphorylated mutant Myt1 completely abolishes 1-MdAde-induced meiotic reinitiation [86]. Thus, the direct phosphorylation of Myt1 by Akt reverses the balance between Myt1 and Cdc25 activities and initiates the activation of cyclin B-Cdc2 to cause meiotic G2/M-phase transition.

A recent study suggests that Akt can also regulate the progression of S to G2 phase *via* phosphorylation of Cdk2. There is a transient nucleocytoplasmic shuttling of Akt during late S and G2 phases [87]. Nuclear Akt phosphorylates Cdk2 at Thr39 which causes temporary cytoplasmic localization of cyclin A/Cdk2 complex. The cytoplasmic redistribution of Cdk2 is required for cell cycle progression from S to G2/M phase, as nonphosphorylated Cdk2 T39A mutant severely affects cell cycle progression. In addition to its role in cell cycle progression, Akt-mediated phosphorylation and cytoplasmic translocation of Cdk2 is also important for apoptosis induced by stresses such as methotrexate and docetaxel [87]. Phosphorylated Cdk2 is sequestered in the cytoplasm and directed to different cytoplasmic substrates including p53, ultimately leading to cell death [87, 88].

## 3. Role of Akt in Genome Stability

### 3.1. Akt Regulation in Response to DNA Damage

**Figure 2:**
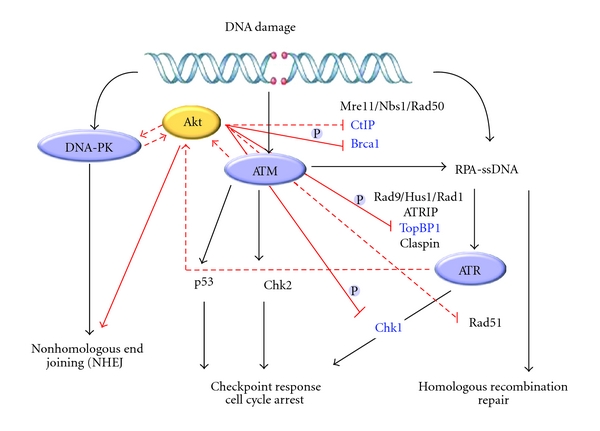
Crosstalk between Akt and DNA damage signalling pathways. Akt can be activated in response to DNA damage through the action of the PI3 kinase-like kinases (PIKKs) ATM, ATR, and DNA-PK. Conversely, active Akt can promote DNA repair *via* NHEJ and inhibit checkpoint signalling and repair *via *recombination through multiple mechanisms and targets. Please see text for additional explanation.

As depicted in Figure 2, Akt is activated not only by growth factors but also by DNA damage [18, 89]. The PIKK family members ATM, ATR, and DNA-PK are involved in Akt activation in response to genotoxic stresses, which may provide a prosurvival signal by triggering cell cycle arrest or inhibiting apoptosis [90–93].

In vertebrates, ATM is recruited to and activated primarily at DNA double-strand breaks in conjunction with MRN (Mre11 : Rad50 : NBS1) complex. Once activated, ATM triggers the phosphorylation of multiple substrates at the site of damage, such as H2AX, Chk2, and tumor suppressor p53, leading to cell cycle arrest, DNA repair, or apoptosis. Mutation of the ATM-gene results in Ataxia telangiectasia (AT), a rare human disease characterized by growth retardation, severe immunodeficiency, insulin resistance, hypersensitivity to radiation, and predisposition to cancer [94]. Interestingly, Akt knockout mice show some similar phenotypic abnormalities to ATM-deficient mice, such as growth retardation, insulin resistance, hypersensitivity to gamma-irradiation, and immunodeficiency [8, 11, 95], indicating that ATM and Akt may be involved in the same signalling transduction pathway. Viniegra et al. provide clear evidence that ATM acts as an upstream activator of Akt in response to insulin or *γ*-radiation [91]. Overexpression of exogenous ATM and Akt in Cos cells promotes Akt Ser473 phosphorylation by insulin or IR and the phosphorylation depends on the PI3K-like catalytic domain of ATM. Although ATM binds to Akt directly, there is no apparent direct phosphorylation of Akt Ser473 by ATM, suggesting that other intermediate proteins downstream of ATM may be involved in direct phosphorylation of Akt in response to insulin or IR. Interestingly, another study by Caporali et al. demonstrates that ATR, but not ATM, is required for Akt phosphorylation at Ser473 in response to temozolomide (TMZ), a therapeutic methylating agent [93]. siRNA-mediated knockdown of ATR but not ATM expression results in abrogation of TMZ-induced phosphorylation of Akt [93]. However, there is no clear evidence that ATR phosphorylates Akt directly in response to TMZ. It is possible that ATR mediates activation of another kinase to phosphorylate Akt.

It has been proposed that DNA-PK (DNA-dependent protein kinase) is also involved in Akt activation in response to DNA damage such as IR and doxorubicin [92]. This pathway is specific for DNA damage as DNA-PK is dispensable for Akt Ser473 phosphorylation upon insulin or growth-factor stimulation [92]. Interestingly, phosphorylated Akt forms nuclear foci and colocalizes with DNA-PK at double-strand DNA breaks and the interaction between Akt and DNA-PK is DNA damagedependent [92]. Akt1 knockout mice resemble the DNA-PK deficiency radiosensitivity phenotype, with attenuated p21^Cip1^ expression and increased apoptosis in response to DNA damage, supporting the notion that DNA-PK/Akt1 pathway has a marked impact on cell survival after DNA damage due to transcriptional regulation of p21^Cip1^ [92]. Beside Akt1, Akt2 and DNA-PK are also required for the stabilization of p53 in response to IR, which is mediated by the inactivation of GSK-3 [96]. The above results suggest a novel mechanism through which DNA-PK phosphorylates and activates Akt at the site of DNA double-strand breaks. This process triggers the induction of a transcriptional program that promotes cell survival in response to DNA damage. Whether DNA-PK directly phosphorylates Akt itself or acts through PDK1 and whether DNA-PK and Akt interact directly remain unclear [97, 98].

### 3.2. Akt Modulates DNA Damage Checkpoint Signalling and DNA Repair

Mounting evidence implicates the role of Akt in modulating checkpoint responses and DNA repair. Activation of Akt overcomes DNA-damage-induced G2 arrest and apoptosis in a p53-independent manner [80, 99–102]. Overexpression of a constitutive active form of Akt or loss of PTEN abrogates G2 cell cycle checkpoint and Chk1 activation upon exposure to genotoxic stresses [80]. In addition, Akt-mediated suppression of G2 arrest is associated with reduced recruitment of Chk2 to sites of DNA damage and inhibition of Chk2 activation in human glioblastoma cells [103]. The ability of Akt to suppress G2 arrest induced by disparate agents suggests that Akt has a broad ability to override this checkpoint regardless of the pathway by which the process is initiated [103].

As the most hazardous DNA lesions, DNA double-strand breaks (DSBs) can lead to genome rearrangements and cell death following exposure to genotoxic stresses. Two mechanisms are primarily involved in repair of DSBs: nonhomologous end joining (NHEJ) and homologous recombination repair (HRR) [104, 105]. NHEJ is evolutionarily conserved in all kingdoms of life and is the predominant DSB repair pathway throughout the cell cycle [106–108]. HRR is a more accurate repair mechanism but restricted to S and G2 phases [109, 110]. Numerous studies have shown that inhibition of Akt activity impairs DSB repair [111–115]. Specific Akt inhibitors or AKT1 siRNA markedly reduce radiation-induced DNA-PKcs phosphorylation at T2609 and S2056, modifications required for activation of DNA-PKcs [116, 117]. Akt1 physically associates with DNA-PKcs, indicating that Akt1 may function as a signalling kinase relevant for DNA-PKcs activation and NHEJ repair [111–115]. It has been shown in malignant glioblastoma that EGFR/ERK/Akt signalling promotes *γ*H2AX foci resolution and enhances both NHEJ and HR repair by modulating the localization, expression, and phosphorylation of DNA-PKcs, ATM, and Rad51. Inhibition of either Akt or MEK/ERK results in significant reduction of NHEJ [115]. These results strongly argue that both Akt and MEK/ERK signalling are critical effectors downstream of EGFR that modulate DNA repair and may contribute to radioresistance in malignant glioblastoma with deregulated EGFR/ERK/Akt signalling. Interestingly, recent studies show that DNA-PKcs acts as an upstream signal for Akt phosphorylation and activation in response to genotoxic stresses [92, 96, 118], suggesting that Akt and DNA-PKcs are tightly coregulated in both checkpoint response and DNA repair.

The activation of HRR in S and G2 phases regulates the accurate repair of DNA lesions, but unregulated HRR could result in amplification of repeated genomic sequences and loss of heterozygosity [119, 120]. In contrast to the positive role of Akt in modulating NHEJ repair, however, recent studies reveal that oncogenic Akt promotes genome instability by repressing HRR under pathologic circumstances. In breast cancer cell lines with high level of Akt1 activity, IR-induced Brca1 and Rad51 foci formation and HRR are strongly impaired compared to cells with low Akt1 activity [121]. Biopsies of sporadic breast cancer patients show strong correlation of Akt1 activation with cytoplasmic Brca1 and Rad51 localization [121]. In addition, active Akt1 induces supernumerary centrosomes and aneuploidy in hamster ovary cells [121].

The above results indicate a novel oncogenic function for Akt1 by producing genomic instability as a consequence of HRR repression, which may play an important function in the pathology of sporadic breast and ovarian cancer [121]. Recent studies by us also provide additional evidence and insight into mechanisms of genomic instability resulting from high level of active Akt due to loss of PTEN. In HCT116 cells ectopically expressing constitutively active Akt or lacking PTEN, both DNA-damage-induced RPA, CtIP and Rad51 foci formation and Chk1 activation are markedly suppressed. Conversely, inhibition of Akt by either selective chemical inhibitors or Akt siRNA restores DNA-damage-induced recruitment of RPA, CtIP, and Rad51 and Chk1 activation. Thus, the inhibitory effect of Akt on ATR-Chk1 signalling and HRR could be due to suppression of DNA damage processing [122].

### 3.3. Akt Targets Involved in DNA Damage Responses

**Figure 3:**
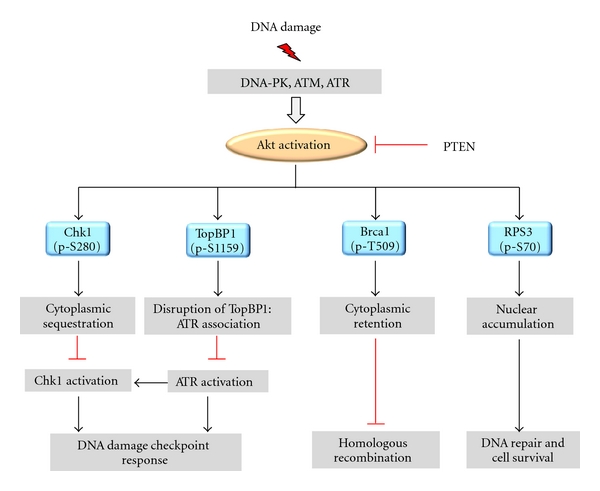
Molecular mechanisms and targets for Akt-mediated genomic instability. Akt can inhibit DNA repair, recombination, and checkpoint signalling by phosphorylating key regulatory proteins such as Chk1, TopBP1, Brca1, and RPS3 at specific sites. Please see text for additional explanation.

Akt is thought to phosphorylate up to 100 substrates, some of which have been implicated in DNA damage checkpoint response and DNA repair. For instance, direct phosphorylation of cell cycle checkpoint kinase Chk1, TopBP1, Brca1, and RPS3 by Akt is important for the activation of DNA damage signalling cascade [101, 123–125] (Figure 3). In addition, Akt activity is pivotal for the focal accumulation of key factors regulating DSB resection and DNA repair, including Rad51, Brca1, CtIP, and RPA [100, 121, 122, 126].

#### 3.3.1. Chk1

As a key checkpoint kinase, Chk1 is activated by a wide range of genotoxic stresses. Activated Chk1 is essential for G2/M and replication checkpoint proficiency when DNA is damaged or replication is incomplete [127–130]. Chk1 is directly phosphorylated by Akt at Ser280, a modification that results in cytoplasmic sequestration [79, 99, 101, 123]. Ser280 phosphorylated Chk1 covalently associates with ubiquitin, ubiquitination of Chk1 does not alter the protein stability; however, it promotes enhanced cytoplasmic localization and inhibition of checkpoint function [101, 123]. The increased cytoplasmic distribution of Chk1 is also seen in primary breast carcinomas tissues with elevated Akt phosphorylation and reduced PTEN level [101, 123]. Beside regulating the subcellular localization, Akt phosphorylation also inhibits the physical interaction between Chk1 and Claspin, which results in the inhibition of Chk1 activity and checkpoint proficiency [124]. By contrast, a recent study by Tonic et al. questions the significance of Chk1 S280 phosphorylation on the checkpoint function, since overexpression of exogenous Chk1 S280E mutant does not override radiation-induced G2 arrest [100]. This apparent discrepancy may be attributable to variable basal levels of active Akt in different cell lines.

#### 3.3.2. TopBP1

 DNA topoisomerase 2-binding protein 1 (TopBP1) is an evolutionally conserved BRCT domain-rich protein that plays a critical role in DNA replication, DNA damage checkpoint responses, and transcriptional regulation [131]. In response to genotoxic stresses, TopBP1 colocalizes and interacts with ATR/ATRIP and promotes phosphorylation of Rad9 in the 9-1-1 (Rad9-Rad1-Hus1) clamp. The C-terminal ATR-activating domain of TopBP1 then stimulates the kinase activity of ATR [132, 133]. Akt phosphorylates TopBP1 at Ser1159, a consensus sequence conserved in human, mouse, rat, and *Xenopus*. This modification induces oligomerization of TopBP1 through its seventh and eighth BRCT domains, and this self-association is crucial for its interaction with E2F1 and transcriptional repression [134]. Ser1159 phosphorylation may also prevent association of ATR with TopBP1 after DNA damage, thus inhibiting activation of ATR and G2/M checkpoint proficiency [124].

#### 3.3.3. Brca1

The tumor suppressor and breast cancer susceptibility gene 1 (Brca1) has been shown to play an integral role in regulating DNA repair, cell cycle checkpoints, transcription, and maintenance of genomic stability. As one of the key players regulating DSB repair, Brca1 forms nuclear foci in response to DNA damage where it colocalizes with Rad51 and facilitates Rad51 filament formation to initiate HR repair. It has been reported recently that oncogenic Akt1 can repress HRR by inducing cytoplasmic retention of Brca1 in human cancer cells [100, 121]. Importantly, cytoplasmic retention of Brca1 is strongly correlated with activated Akt1 in biopsies from sporadic breast cancer patients [121]. Although Akt is able to phosphorylate Thr509 of Brca1 within the nuclear localization sequences (NLS), there is no direct evidence that Thr509 phosphorylation is required for cytoplasmic localization of Brca1 [135–137]. One potential scenario could be that Thr509 phosphorylation prevents Brca1 interacting with other proteins that localize to DNA damage foci. Conversely, Brca1 could also regulate Akt pathway in a negative manner. Brca1 interacts with phospho-Akt through Brca1-BRCT domains leading to its ubiquitination and degradation, thereby downregulating Akt signalling [138]. Thus, elucidation of the precise molecular functions of Brca1-Akt pathway will be particularly important to understand tumorigenesis of hereditary and sporadic breast cancer.

#### 3.3.4. RPS3

Ribosomal protein S3 (RPS3) is a component of the 40S ribosomal subunit which is required for ribosome biogenesis [139]. Apart from its ribosomal functions, RPS3 is also known as UV endonuclease III which participates in the cleavage of DNA lesions induced by UV irradiation. RPS3 has apurinic/apyrimidinic (AP)^2^ endonuclease activity functioning in DNA repair at the 3′ of the AP sites after DNA damage [140–142]. A recent study by Lee et al. demonstrates that RPS3 is a physiological target of Akt and a novel mediator of neuronal apoptosis. Akt physically binds to and phosphorylates RPS3 at Thr70. Akt-dependent phosphorylation suppresses the proapoptotic function of RPS3 by governing its nuclear accumulation and enhancing its endonuclease activity [125]. It is conceivable that Akt promotes cell survival after DNA damage by regulating the DNA repair activity of RPS3 [143, 144]. Manipulation of nuclear RPS3 and its Akt-mediated phosphorylation might provide a new dimension for therapeutics of neurological diseases.

Several lines of evidence demonstrate the role of Akt in manipulating the subcellular localization or expression of the key factors regulating checkpoint response and DNA repair. Overexpression of constitutive active form of Akt or loss of PTEN disrupts the nuclear foci formation of DNA-PKcs, Rad51, Brca1, CtIP and RPA in response to DNA damage [121, 126]. The cytoplasmic retention of Brca1 and Rad51 also correlates with activated Akt1 in sporadic breast cancer tissues [121, 126]. In addition, it has been proposed that Akt activity could regulate the expression of Mre11 through GSK3*β*/*β*-catenin/LEF-1 pathway, which promotes the repair of radiation-induced DSBs in both CNE2 and HeLa cells [145]. The PI3K/Akt signalling pathway also acts together with MKK1/2-ERK1/2 to control Rad51 expression and protein stability in human non-small-cell lung cancer cells (NSCLCs) [146, 147].

## 4. Implications for Cancer Development and Therapy

Akt signaling is frequently activated in human cancers due to genetic and epigenetic alterations and has a role in neoplastic transformation [148–152]. Although Akt gene mutations are not widely reported, the amplification, overexpression, and activation of Akt occurs at high frequency in a number of human cancers [148, 150]. Alterations of Akt signalling in human cancer also result from mutations of the upstream PI3K kinase and PTEN. PTEN is one of the most frequently mutated genes, second only to p53 in human cancers [30, 153]. Inactivation of PTEN is strongly correlated with the activation of Akt which in turn controls tumor cell proliferation. PTEN activity is lost by mutation, deletion, or promoter methylation silencing at high frequency in many primary and metastatic human cancers [154–156]. Many activating mutations have also been described in PI3KCA gene, upstream of Akt, which encodes one of the p110*α* catalytic subunits of PI3K. PI3KCA mutations (E542K, E545K, H1047R) are frequently observed in human colon, gastric, breast, and lung cancers and glioblastoma. They are able to constitutively activate Akt and enhance its oncogenic activity [157–162].

The early stages of neoplasia are often associated with spontaneous genotoxic stresses resulting in DNA damage. If the damage is not repaired, DNA injury can result in mutation of tumor suppressor genes and activation of oncogenes, triggering uncontrolled cell proliferation and genome instability, the major cause of cancer. Inherited mutations that affect DNA repair genes are associated with human cancers [105, 163–166]. However, the majority of cancers are sporadic; thus, understanding the mechanisms of tumorigenesis will be particularly important for cancer therapy. Recently, mounting evidence indicate that Akt signalling pathway plays a key role in modulating DNA damage response and genome stability in complex ways.

Activated Akt may contribute to resistance to chemo- or radiotherapy by stimulating NHEJ repair and promoting cancer cell survival. Several considerations argue that this may be the case. Firstly, the bulk of DSBs are repaired by NHEJ which is active throughout the cell cycle and DNA-PK is required for NHEJ repair. It has been shown that Akt physically associates with DNA-PKcs and activates it by phosphorylation [113]. In addition, DNA-PKcs could activate Akt in response to genotoxic stresses [92]. Therefore this positive feedback loop could contribute to highly DNA repair proficiency and chemo- or radioresistance in some tumor cells with constitutive Akt activation. Secondly, oncogenic Akt may contribute to genomic instability *via* abrogation of cell cycle checkpoints, suppression of Chk1 activation and HRR, as well as induction of supernumerary centrosomes and aneuploidy. Taken together, activation of Akt pathway potentially contributes to tumorigenesis and tumor cell survival through multiple mechanisms. Future work will seek to evaluate the precise role of Akt in regulating these processes.

Over the past decade numerous studies have accumulated supporting the idea that inhibition of PI3K/Akt pathway leads to radiosensitization in glioblastoma and various carcinomas, including colon, bladder, prostate, head and neck, and cervix [167–175]. The combination of PI3K inhibitor and radiation causes an increase in apoptosis and a decrease in clonogenic survival, whereas expression of constitutively active Akt blocks apoptosis induced by radiation and prevents radiosensitization by PI3K inhibitor, indicating that activated Akt promotes cell survival through inhibition of IR-induced apoptosis [167–175]. Akt activity also promotes resistance to chemotherapy in breast cancer and ovarian cancer cell lines [176, 177]. When combined with therapies commonly used in breast cancer treatment, the PI3K inhibitor enhances apoptosis caused by doxorubicin, trastuzumab, paclitaxel, etoposide, or tamoxifen, suggesting that inhibition of Akt in breast cancer patients may increase the efficacy of these therapies [176, 177]. Loss of PTEN and the presence of active Akt are correlated with poor differentiation, lymph node involvement, distant metastasis, and late stage in NSCLC patients. Preclinical data suggest that modulation of Akt activity sensitizes cells to chemo- or radiotherapy [89, 178]. The combinations of drugs that modulate Akt activation with the therapeutic modalities used in NSCLC are thought to improve cancer treatment [150].

The PI3K/Akt/PTEN pathway therefore emerges as one of most promising targets of anticancer drugs in the immediate future. Currently, clinical trials with inhibitors of Akt in monotherapy or combination with other anticancer drugs are underway in cancer patients [150, 179] (Table 1). There are several promising candidate Akt inhibitors, such as lipid-based inhibitors that bind to the PH domain, ATP-competitive and noncompetitive small molecule inhibitors, and peptide-based inhibitors [150, 180, 181]. Perifosine (Keryx Biopharmaceuticals, New York, NY, US) is a lipid-based inhibitor which can inhibit the translocation of all Akt isoforms to the membrane by interacting with the PH domain [182, 183]. In both preclinical and early clinical trials, perifosine displays antiproliferative effects in many tumor cell lines and sensitizes tumor cells to radiation and chemotherapy *in vitro* [183, 184]. Phase II clinical trials with perifosine combined with radiotherapy, chemotherapy or other anticancer agents are ongoing [150, 180]. MK-2206 (Merck and Co., Whitehouse Station, NJ, US) is an allosteric Akt inhibitor, which induces a conformational change in the Akt protein to inhibit both the activity and activation of the kinase and blocks signal transduction through the PTEN pathway [185, 186]. Preclinical studies demonstrate that MK-2206 exerts more potent tumor inhibitory activities when in combination with other targeted therapies (erlotinib, lapatinib) or cytotoxic agents such as doxorubicin, docetaxel, and 5-fluorouracil [187]. An oral phase I trial with MK-2206 in patients with advanced solid tumors was just completed [179, 188]. Currently, over 10 enrolling phase II clinical trials with MK-2206 or combined with chemotherapy or targeted anticancer therapy are ongoing in multiple tumor types (http://clinicaltrials.gov/ct2/results?term=MK-2206). Other orally dosed ATP competitive Akt inhibitors undergoing phase I trials include GSK2141795 (GlaxoSmithKline, Brentford, UK) and GSK2110183 [189]. A potent ATP-competitive inhibitor GSK69069 shows strong efficacy in xenograft models from ovarian, prostate, and breast carcinoma cell lines [150]. It is currently in clinical development as an intravenous agent used in patients with solid tumors or haematological malignancies [189]. The search for small molecule inhibitor and peptide-based inhibitors is a new frontier in drug discovery. Akt antisense inhibitors are also being developed. RX-0201 represents an antisense oligonucleotide to Akt1. A phase II ongoing clinical trial is assessing the combination efficacy of RX-0201 and gemcitabine in metastatic pancreatic cancer [179, 190].

Beside that, inhibitors targeted upstream or downstream of Akt such as PI3K and mTOR are more advanced in their development than Akt inhibitors. Several PI3K and mTOR inhibitors are in preclinical and phase II clinical trials at present [189, 191]. Regarding the complexity of PI3K/Akt/PTEN/mTOR signalling in different types of tumors, combinations of targeted therapeutics might be required for efficacy and safety for cancer treatment in the future. 

## Figures and Tables

**Table 1:** Akt inhibitors currently in clinical trials.

Compound	Description	Phase	Tumor
MK-2206	Allosteric kinase inhibitor	I	Metastatic solid tumors, non-small-cell lung cancer, advanced solid tumors, breast cancer, leukemia, lymphoma, neoplasms malignant, kidney cancer, prostate cancer, brain and central nervous system tumors, small intestine cancer
		II	Ovarian cancer, fallopian tube and primary peritoneal cancer, leukemia, lymphoma, colorectal neoplasms, breast cancer, esophageal cancer, gastric cancer, endometrial cancer, prostate cancer, recurrent nasopharyngeal carcinoma, head and neck cancer, kidney cancer, lung cancer, extrahepatic bile duct cancer, gallbladder cancer, liver cancer

Perifosine	Lipid-based allosteric kinase inhibitor	I	Pediatric solid tumors, multiple myeloma, solid tumors, colon cancer, renal cancer, ovarian cancer, neoplasms, brain and central nervous system tumors, leukemia, lymphoma
		II	Malignant gliomas, CNS, brain cancer, Waldenstrom's macroglobulinemia, lymphomas, colon cancer, gastrointestinal stromal tumors, multiple myeloma, chondrosarcomas, alveolar soft part sarcomas, kidney cancer, renal cell carcinoma, leukemia, lymphoma, brain and central nervous system tumors, melanoma (skin), endometrial cancer, sarcoma, prostate cancer, head and neck cancer, pancreatic cancer, breast cancer
		III	Multiple myeloma, colorectal cancer

GSK-2141795	ATP competitive inhibitor	I	Ovarian cancer, solid tumors, and lymphoma

GSK-2110183	ATP competitive inhibitor	I	Multiple myeloma, hematologic malignancies
		II	Langerhans cell histiocytosis, hematologic malignancies

RX-0201	interfering RNA	II	Metastatic pancreatic cancer
